# Integrating bioinformatics analysis, machine learning, and experimental validation to identify pyroptosis-related genes in the diagnosis of sepsis combined with acute liver failure

**DOI:** 10.1186/s41065-025-00522-4

**Published:** 2025-08-08

**Authors:** Jing Yan, Yifeng Pan, Chaoqi Chen, Lijian Liu, Jinjing Tan, Juan Li, Liqun Li, Sheng Xie

**Affiliations:** 1 Graduate School of Guangxi University of Chinese Medicine, Nanning, 530200 Guangxi China; 2 The Eighth Clinical Medical College of Guangzhou University of Chinese Medicine, Foshan, 528000 Guangdong China; 3 The First Affiliated Hospital of Guangxi University of Chinese Medicine, Nanning, 530001 Guangxi China

**Keywords:** Sepsis, Acute liver failure, Pyroptosis, Immune cell infiltration, Animal experimental validation

## Abstract

**Background:**

Sepsis is frequently combined with acute liver failure (ALF), a critical determinant in the mortality of septic patients. Pyroptosis is a significant form of programmed cell death that plays an important role in the inflammatory response. Research has been conducted to elucidate the relationship between pyroptosis, sepsis, and ALF, but the mechanism of action remains unclear.

**Methods:**

Datasets relating to sepsis and ALF were obtained from the Gene Expression Omnibus (GEO). The intersection of differentially expressed genes (DEGs) and pyroptosis-related genes for sepsis and ALF was identified. Simultaneously, a gene diagnosis model for sepsis and ALF was developed using machine learning, and the model’s accuracy was assessed through the plotting of the ROC curves and confusion matrix. The Hub genes identified by the model with an area under the curve (AUC) value ≥ 0.7 were used for the investigation of immune cell infiltration to explain the molecular mechanism of sepsis combined with ALF. The precise mechanism of action of these model genes in sepsis combined with ALF was evaluated through animal experiments.

**Results:**

Machine learning revealed that GABARAP and ITCH may serve as diagnostic biomarkers for pyroptosis in sepsis combined with ALF. The examination of immune cell infiltration indicated that immune dysregulation is present in both sepsis and ALF and preliminarily suggested that GABARAP and ITCH may be pivotal in cellular immunity responses, particularly those mediated by T cells. Animal experiments further validated that in the process of sepsis combined with ALF, the expression level of GABARAP is elevated, while the expression level of ITCH is diminished.

**Conclusions:**

We found GABARAP and ITCH may serve as diagnostic biomarkers for pyroptosis in sepsis combined with ALF, suggesting their potential involvement in the initiation and advancement of sepsis combined with ALF through cellular immunomodulatory pathways.

**Clinical trial number:**

Not applicable.

**Supplementary Information:**

The online version contains supplementary material available at 10.1186/s41065-025-00522-4.

## Introduction

Sepsis is a systemic inflammatory reaction condition triggered by infection, frequently resulting in multi-organ dysfunction or failure, significantly endangering human life and health, and has emerged as a crucial challenge in the realm of intensive care medicine [1]. A meta-analysis showed that the incidence of ICU sepsis was around 58 per 100,000 person-years, with a hospitalization mortality rate of 41.9% among patients [2]. Between 2017 and 2019, the yearly standardized incidence rate of sepsis in China rose from 328.25 to 421.85 cases per 100,000 person-years, with the fatality rate escalating annually [3]. Acute liver failure (ALF) is a collection of clinical syndromes marked by significant liver impairment over a brief duration, primarily presenting with jaundice, hepatorenal syndrome, and hepatic encephalopathy [4], and historically, it has had a fatality rate reaching 80% [5]. The prevalence of ALF exceeds 1 million cases annually on a global scale, markedly augmenting the burden of disease worldwide [6].

Recent studies increasingly indicate a strong correlation between sepsis and ALF. The prevalence of ALF in sepsis patients reaches 22% [7]. The early onset of ALF is an significant potential factor for worse outcomes among individuals with sepsis [8]. Specific investigations have shown a positive correlation between sepsis and the severity of ALF [9]. Sepsis and ALF are associated with the pathophysiology of immune system dysregulation, inflammatory cytokine storms, and pyroptosis. Nevertheless, the etiology of comorbidity between the two conditions remains unclear, and efficacious pharmacological interventions are absent for their management. While antibiotics are the preferred pharmacological agents for sepsis treatment, prolonged use of them may impose an additional strain on the liver and potentially induce ALF or make it worse [10]. Conversely, certain medications for ALF, including glucocorticoids, can significantly reduce patient symptoms; however, they may also precipitate immune dysfunction and elevate the risk of infectious illnesses such as sepsis by intensifying inflammatory responses [11]. In conclusion, the pathophysiological mechanism of sepsis combined with ALF remains inadequately understood, presenting a significant obstacle to clinical treatment.

Pyroptosis is a type of programmed cell death, typically initiated by inflammasomes, that is crucial for sustaining host defense against pathogen infections [12]. Pyroptosis is commonly observed in immune cells, including neutrophils, dendritic cells, natural killer cells, and T cells [13]. Research shows that pyroptosis plays a key role in the progression of sepsis [14]; exosome miR-30d-5p facilitates macrophage polarization and initiates pyroptosis, consequently advancing the onset of sepsis and its associated complications [15]; elevated levels of pyroptosis have been observed in ALF mice [16]; and the activation of cellular pyroptosis driven by the NLRP3 inflammasome worsens acute hepatic injury linked to sepsis during the inflammatory response [17]. Emerging findings show pyroptosis has the potential to significantly influence cellular immune responses [13, 18]. For instance, pyroptosis relates to the regulation and activation of CD8 + T cells and NK cells [19] and facilitates the metabolism and differentiation of inflammatory immune cells (such as neutrophils) [20]. The evidence presented indicates that pyroptosis and cellular immunity may play a substantial role in the pathophysiology of sepsis and ALF. Therefore, the advancement of co-diagnostic biomarkers aimed at pyroptosis and cellular immunity to clarify the shared biological mechanisms of sepsis and ALF constitutes a crucial approach to tackling the challenges involving sepsis combined with ALF.

This study involved the integration of bioinformatics analysis, microarray data, and machine learning, resulting in the identification of essential diagnostic markers of pyroptosis in sepsis combined with ALF. We employed CIBERSORT to clarify the relationship between these crucial biomarkers and immune cell infiltration and conducted experimental validation to determine the importance of these core genes in sepsis combined with ALF, eventually clarifying the biological mechanisms underlying the disease.

## Materials and methods

### Study design

**Fig. 1:**
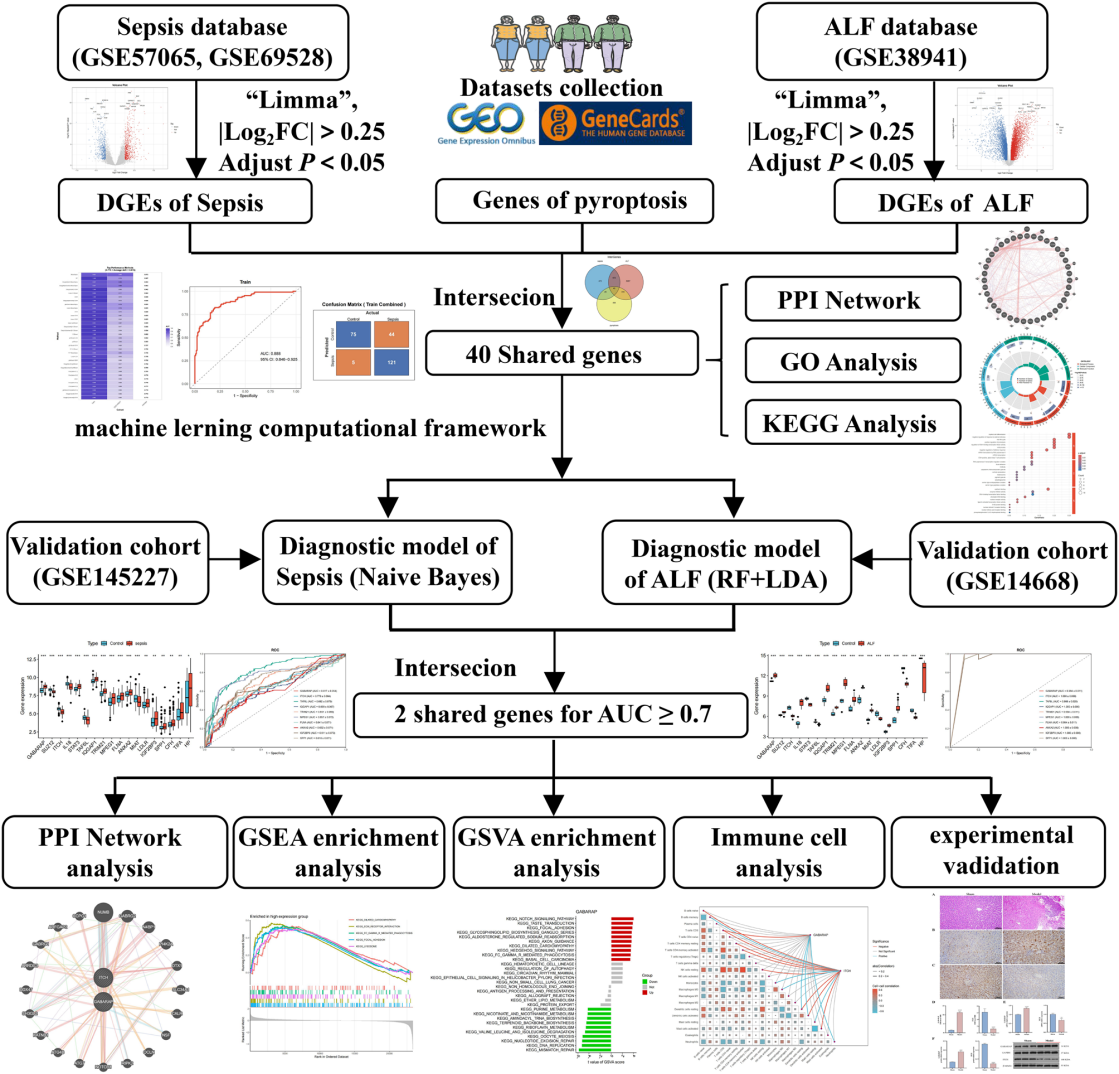
Flow chart of investigation

The study’s general design is illustrated in Fig. 1. Initially, we acquired the sepsis and ALF datasets from the Gene Expression Omnibus (GEO) (http://www.ncbi.nlm.nih.gov/geo). The differentially expressed genes (DEGs) associated with these two conditions were intersected with the pyroptosis genes acquired from GeneCards (https://www.genecards.org). The resulting intersected genes were employed for protein-protein interaction (PPI) network analysis, gene ontology (GO) enrichment analysis, and Kyoto Encyclopedia of Genes and Genomes (KEGG) analysis. Simultaneously, machine learning was conducted for sepsis and ALF utilizing intersection genes to develop a genetic diagnostic model, with the model’s accuracy evaluated using ROC curves and confusion matrices. The gene diagnostic models for sepsis and ALF were analyzed as intersections. Box plots, correlation plots, and ROC curves were produced for the modeled genes to discover Hub genes with an area under the curve (AUC) value ≥ 0.7. Subsequently, we performed protein-protein interaction (PPI) network analysis, Gene Set Variation Analysis (GSVA), Gene Set Enrichment Analysis (GSEA), immune cell infiltration analysis, and animal experiments for validation, consequently clarifying the molecular mechanism of sepsis combined with ALF. The data utilized in this study were sourced from open databases, negating the necessity for reapplication for clinical ethics approval.

### Data sources

Gene expression profiles related to sepsis and ALF were acquired from GEO. Selection criteria for the microarray datasets: (a) the organism was Homo sapiens; (b) arrays were used for gene expression profiling; (c) microarray datasets comprised case and control groups; (d) a minimum of six participants per dataset, with at least three individuals in each group. And genes related to pyroptosis were acquired from GeneCards.

### Evaluation for DEGs

The gene expression profiles of sepsis and acute liver failure were annotated and pre-processed using R software (version 4.3.3). To guarantee data quality, we eliminated gene probes with missing values and non-specific probes, and then the data was normalized with the “limma” package and its function “normalizeBetweenArrays” [21]. The GSE57065 and GSE69528 datasets were amalgamated, and batch effects were mitigated utilizing the “sva” package, with findings displayed as box plots and principal component analysis (PCA) cluster plots [22]. The DEGs of sepsis and ALF were identified using the “limma” package, volcano plots of DEGs were generated using the “pheatmap” package, and heatmaps of DEGs were created for visual analysis using the “ggplot2” package. During the identification of DEGs, the criteria were established at *P* < 0.05 and|Log_2_FC| ≥ 0.25. Log_2_FC values exceeding 0.25 indicated up-regulated genes, while those below − 0.25 signified down-regulated genes.

### Functional enriched analysis of DEGs

The DEGs of sepsis, the DEGs of ALF, and the genes related to pyroptosis were intersected, and the intersecting Venn diagrams were generated using R software packages (ggvenn). The PPI network analysis of the intersected genes was conducted using GeneMANIA (https://genemania.org). R software package “clusterProfiler” was used for GO and KEGG enrichment analysis, “enrichplot” was used to visualize the enrichment results, and “ggplot2” was used to construct and optimize the publication-level visual effects [23].

### Machine learning

During the 10-fold cross-validation process, a total of 12 machine learning algorithms were used, as detailed below: Support Vector Machine (SVM) [24], Random Forest (RF) [25], Linear Discriminant Analysis (LDA) [26], Naive Bayes [27], Generalized Linear Model Boosting (glmBoost) [28], Extreme Gradient Boosting (XGBoost) [29], Stepglm [30], Partial Least Squares Regression for Generalized Linear Models (plsRglm) [31], Least Absolute Shrinkage and Selection Operator (Lasso) [32], Gradient Boosting Machine (GBM) [33], Ridge [34], Elastic Net (Enet) [35]. Arrange and combine these 12 machine learning algorithms to form 113 combinations, and develop models based on 10-fold cross-validation to determine the best diagnostic model for the 40 intersecting genes [36]. Subsequently, the performance metrics of the training and test sets were calculated using the R package “pROC” [37], and the ROC curve and confusion matrix were visualized using “ggplot2” to determine the optimal classification threshold for a more intuitive presentation of the model’s performance [38, 39]. Ultimately, the model with higher average AUC values, higher number of modeled genes and better predictive performance in the training and test sets is selected. Supplementary Material S1 provides a detailed description of all the algorithms used in this study, along with important parameters to effectively tune the model of each algorithm.

### Characterization gene evaluation

The external dataset GSE145227 was used to assess the efficacy of models GSE57065 and GSE69528 in differentiating between sepsis and normal controls. Meanwhile, GSE14668 was used to assess the efficacy of model GSE38941 in differentiating between ALF and normal controls. The most accurately predicted genes of sepsis and ALF were intersected and represented in Venn diagrams. The multi-gene box plot was drawn by the R software package “ggpubr” and the statistical significance was marked (*P* < 0.05). The “Performance Analytics” package was used to calculate the Spearman correlation coefficient matrix of the intersection genes, and the scatter plot, histogram and coefficient correlation visualization were integrated to reveal the regulatory relationship between genes. ROC curves were plotted using R packages such as “ggplot2”, “scales”, and “pROC”, and the corresponding AUC values were calculated. The potential application value of intersection model genes in predicting sepsis combined with ALF was evaluated by the ROC curve. AUC ≥ 0.7 indicates that the diagnostic model has moderate clinical value and can be used as the basis for further research [40].

### Hub gene enrichment analysis

The Hub genes with an AUC value ≥ 0.7 were examined using the PPI network via GeneMANIA. Gene enrichment analysis was performed with the R software packages including “limma”, “org.Hs.eg.db”, and “clusterProfiler”. Using the “enrichplot” package to illustrate the five most active and five most suppressed pathways in sepsis and ALF. Enrichment analysis of GSVA and GSEA was conducted using R software packages including “reshape2”, “GSEABase”, and “GSVA“ [41, 42].

### Examination of immune cell infiltration

The infiltration of immune cells in sepsis and ALF was assessed using the R software package “CIBERSORT,” with a significance threshold of *P* < 0.05 for screening [43]. The immune cell infiltration was examined and illustrated utilizing R packages “reshape2”, “ggpubr”, and “corrplot”. The relationship between the Hub genes common to sepsis and ALF and the immune infiltrating cells was examined using the R software package “limma”, “tidyverse”, and “linkET“ [44]. Pearson’s correlation coefficient was employed to assess the relationship between hub genes and immune infiltrating cells.

### Animal experiments

#### Preparation of sepsis combined with ALF model rats

Twenty SD rats, evenly divided between males and females, weighing 160 ~ 200 g, were obtained from Hunan Slake Jinda Laboratory Animal Co. Ltd. (Hunan, China), having the animal production license certificate of SCXK (Hunan) 2019-0004. Before the experiment, they were acclimatized and fed in the SPF-grade animal room of Guangxi University of Chinese Medicine (temperature of 20℃~22℃, humidity 50%~60%) for 7 days, with a light/dark cycle of 12 h and free feeding and drinking intake. Ten rats, evenly divided by sex, were randomly assigned to two groups, the model and sham-operated groups, by using the random number table method. In the model group, the sepsis combined with ALF model was established through cecal ligation and perforation (CLP) [45]. The sham-operated group underwent only the abdominal incision and suturing procedures. All rats were subjected to fasting without water post-surgery. The livers were dissected 48 h postoperatively, and liver histopathological damage was evaluated using hematoxylin and eosin staining (HE staining) to validate the success of the sepsis combined with ALF model. The animal experimentation in this study received approval from the Ethics Committee of Guangxi University of Chinese Medicine (Ethics No. DW20230324-122), and all procedures were performed under the supervision of the Ethics Committee of Guangxi University of Chinese Medicine.

#### HE staining and immunohistochemical analysis

A segment of liver tissue was fixed with 4% paraformaldehyde (BL539A, Biosharp, China) for 24 h, followed by dehydration, embedding, and sectioning. The liver tissue sections were stained using hematoxylin staining solution (ZLI-9610, ZSGB-BIO, China) and eosin staining solution (G1100, Solarbio, China). The pathogenic changes in liver tissues were assessed by orthogonal white light photomicrographic microscopy (BX43, OLYMPUS, Japan). The procedure of immunohistochemistry analysis is as follows: Initially, paraffin-embedded liver tissue slices were dewaxed and hydrated for heat-induced antigen retrieval. The tissues were incubated at ambient temperature for 20 min and subsequently overnight at 4 °C with primary antibodies GABARAP (A12568, Abclonal, China, 1/100) and ITCH (A17389, Abclonal, China, 1/100). Subsequently, PBS washing was conducted.After that, the tissues were incubated at ambient temperature with Goat Anti-Rabbit IgG H&L (HRP) (ZB-2301, ZSGB-Bio, China, 1/100). Then, a PBS wash was performed again, and the pigment was created via DAB. The samples were subsequently dehydrated and sealed. The GABARAP and ITCH protein expression were observered under an orthogonal microscope, with analysis performed using ImageJ (version: 1.8.0.112). Protein expression intensity = Integrated Optical Density (IOD) / Area * 1000.

#### Quantitative reverse transcription polymerase chain reaction (qRT-PCR) detection

Trizon Reagent (CW0580S, CWBIO, China) was utilized for the extraction of total RNA from the sample, while HiScript II Q RT SuperMix for qPCR (+ gDNA wiper) (R223-01, Vazyme, China) was employed for cDNA synthesis. In accordance with the guidelines of the SYBR Premix Ex Taq II Reagent (Q711-02, Vazamy, China), qRT-PCR analysis was conducted using a fluorescence PCR instrument (CFX Connect TM, Bio-Rad, USA). The relative gene expression levels of GABARAP and ITCH were measured using the 2^−ΔΔCT^ technique, with GAPDH serving as the internal control gene. The primer sequences employed are detailed in Table 1, produced by General Biosystems (Anhui) Co., Ltd.

**Table 1:** Primer sequences

Genes	forward primer(5’-3’)	reverse primer(5’-3’)
GAPDH	GACAACTTTGGCATCGTGGA	ATGCAGGGATGATGTTCTGG
GABARAP	CGGATAGGGGACCTGGACAA	TGGCACTGGTGGGTGGAAT
ITCH	TGTTTTATTGGGAACTGCTGG	CTCTGTTGGCTCTTTGTCACCT

#### Western blot analysis

Initially, approximately 50 mg of liver tissue was collected, total protein was extracted, and the BCA Protein Assay Kit (E-BC-K318-M, Elabscience) was employed for protein quantification. The protein concentration of each sample was calculated, followed by protein sample preparation. Then SDS-PAGE electrophoresis was conducted, and membranes were sealed post membrane transfer. Subsequently, the samples were incubated with primary antibodies GABARAP (A12568, Abclonal, 1/1000), ITCH (A17389, Abclonal, 1/1000), and the internal control antibodies GAPDH (T0004, Affinity, 1/10000) and β-tubulin (T0023, Affinity, 1/8000). After, the samples were incubated with the secondary antibody HRP-conjugated Goat Anti-Rabbit IgG (H + L) (GB23303, Servicebio, 1/2000) at 4 °C overnight. Following a 10-second exposure in a darkroom, the film was immersed in the developer for 1 min. It was thereafter rinsed in water once prior to being immersed in the fixer for one minute. The film was later scanned with a scanner (V19, EPSON, Japan), and the gray levels were measured using ImageJ software. The grayscale contrast values between GABARAP and ITCH bands, together with the internal control bands, underwent statistical analysis.

#### Statistical analysis

Animal experimental data were analyzed using SPSS 22.0. Measurement data were represented as mean ± standard deviation ($\:\stackrel{-}{x}\:\pm\:\:s$). The experimental approach was conducted thrice to ascertain the stability and reliability of the results. The One-Way ANOVA test was utilized in the statistical analysis when the data adhered to normal distribution and demonstrated homogeneity of variance; conversely, the Mann-Whitney U test was applied when the data failed to meet the criteria for normal distribution or homogeneity of variance. *P <* 0.05 indicated that the difference was statistically significant.

## Results

### Data preprocessing and differentially expressed genes analysis

**Fig. 2:**
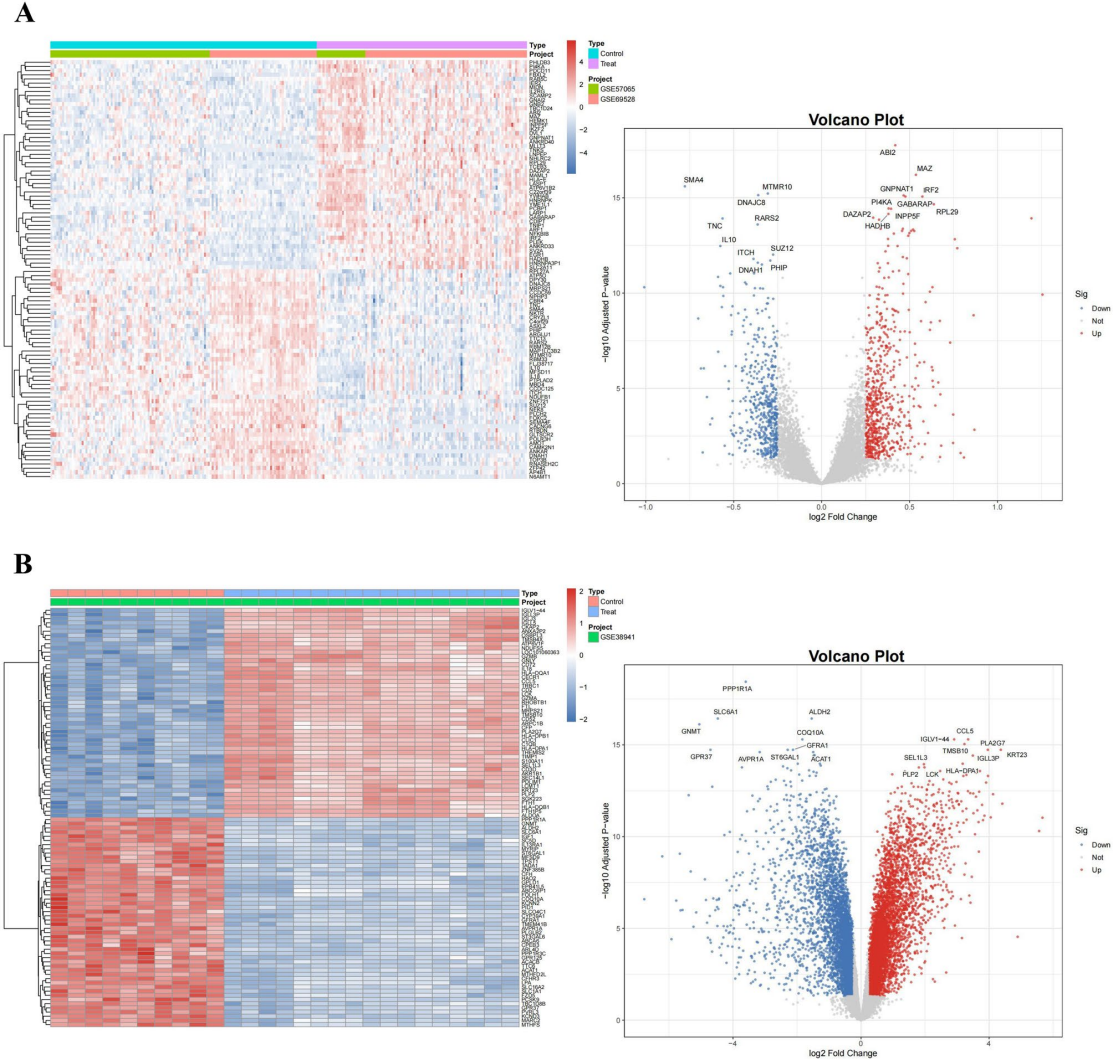
Differential expression analysis of sepsis and ALF. (**A**) Heat map and volcano plot of DEGs in peripheral blood samples of sepsis; (**B**) Heat map and volcano plot of DEGs in liver tissue samples of ALF

Table 2 indicates that sepsis obtained three datasets (GSE57065, GSE69528, GSE145227), while ALF obtained two datasets (GSE38941, GSE14668). Box graphs illustrating the sepsis GSE57065 and GSE69528 datasets prior to and subsequent to concatenated normalization (Supplementary Figure S1A), alongside a PCA analysis cluster plot depicting conditions before and after correction for inter-batch discrepancies (Supplementary Figure S1B). In the sepsis cohort, 1211 DEGs were recognized, comprising 691 up-regulated genes and 520 down-regulated genes (Supplementary Table S1); the heatmap illustrated the top 100 DEGs, while the volcano plot depicted the top 20 up-regulated and down-regulated genes (Fig. 2A). In the ALF cohort, 10,015 DEGs were identified, comprising 5,156 up-regulated genes and 4,859 down-regulated genes (Supplementary Table S2); the heatmap illustrated the top 100 DEGs, while the volcano plot depicted the top 20 up- and down-regulated genes (Fig. 2B).

**Table 2:** Detailed information of datasets

GSE series	Disease	country	Platform file name	Number of case samples	Number of control samples
GSE57065	Sepsis	France	GPL570	82	25
GSE69528	Sepsis	USA	GPL10558	83	55
GSE145227	Sepsis	China	GPL23178	10	12
GSE38941	ALF	USA	GPL570	17	10
GSE14668	ALF	USA	GPL570	8	8

### DEGs function enrichment analysis

**Fig. 3:**
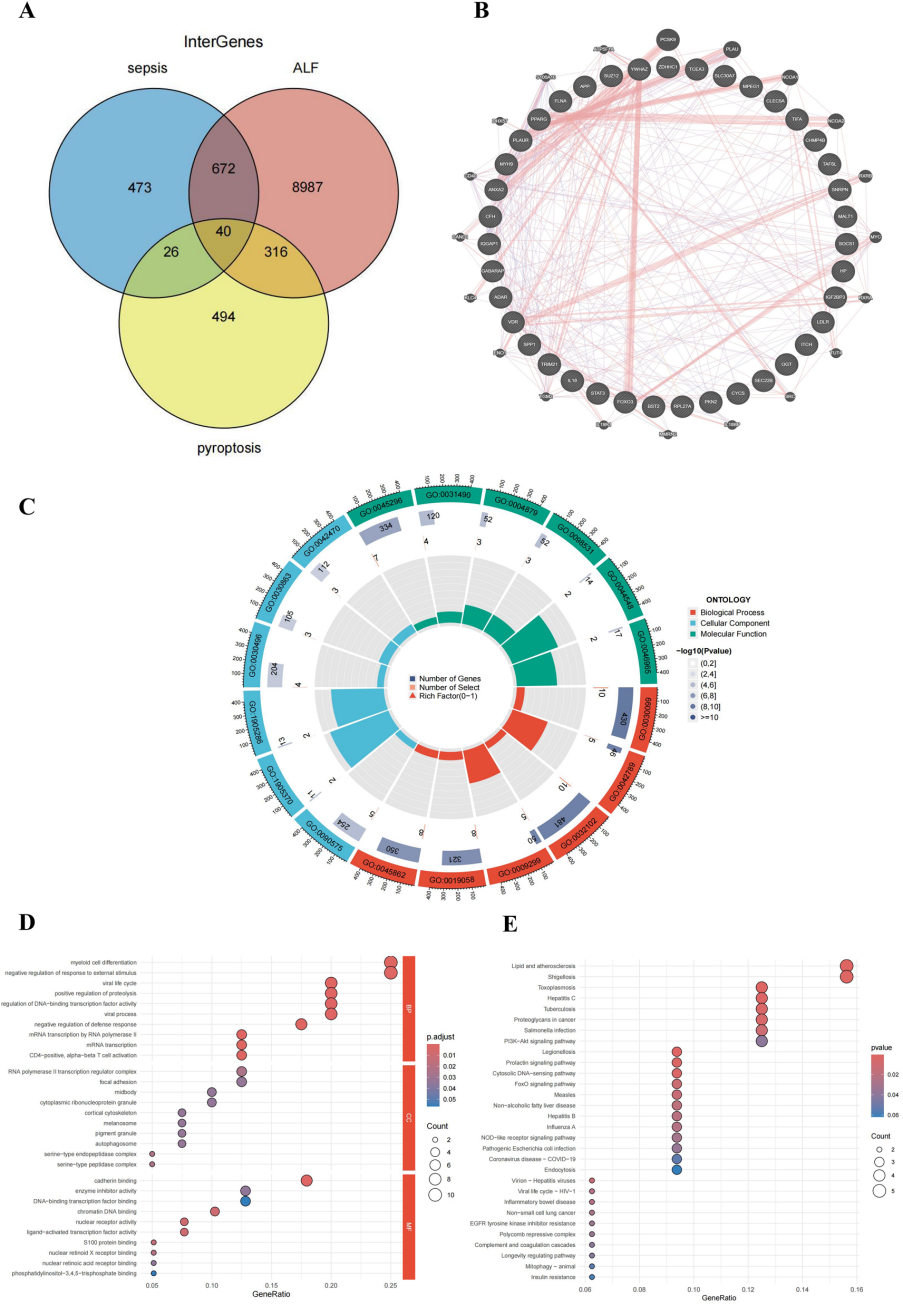
Functional enrichment analysis of DEGs. (**A**) Venn diagram of intersecting genes of sepsis, ALF and pyroptosis; (**B**) PPI network analysis based on GeneMANIA; (**C**) Circle diagram of GO enrichment analysis results; (**D**) Bubble diagram of GO enrichment analysis results; (**E**) Bubble diagram of KEGG enrichment analysis results

The DEGs and pyroptosis genes associated with both sepsis and ALF were intersected, resulting in the identification of 40 intersecting genes (Fig. 3A). The PPI network diagram illustrated the interconnections among these 40 intersecting genes (Fig. 3B), comprising 20 associated genes and 364 edges. The GO enrichment study generated a total of 2,232 GO keywords (Supplementary Table S3). They contain 1,785 biological process (BP) related GO terms, including myeloid cell differentiation (GO:0030099), mRNA transcription by RNA polymerase II (GO:0042789), and positive regulation of proteolysis (GO:0045862); 243 cell component (CC) related terms, including RNA polymerase II transcription regulator complex (GO:0090575), midbody (GO:0030496), and cortical cytoskeleton (GO:0030863); and 204 molecular function (MF) related GO terms, including cadherin binding (GO:0045296), nuclear receptor activity (GO:0004879), and chromatin DNA binding (GO:0031490) (Fig. 3C-D).

A total of 129 enriched pathways were discovered in the KEGG analysis (Supplementary Table S4). The principal pathways are illustrated in bubble diagrams, mostly encompassing the PI3K-Akt signaling pathway, NOD-like receptor signaling pathway, and EGFR tyrosine kinase inhibitor resistance pathway (Fig. 3E). In conclusion, we determined that pyroptosis influences the onset and progression of sepsis combined with ALF through those specific biological processes and pathways.

### Machine learning developed a diagnostic model for sepsis and ALF

**Fig. 4:**
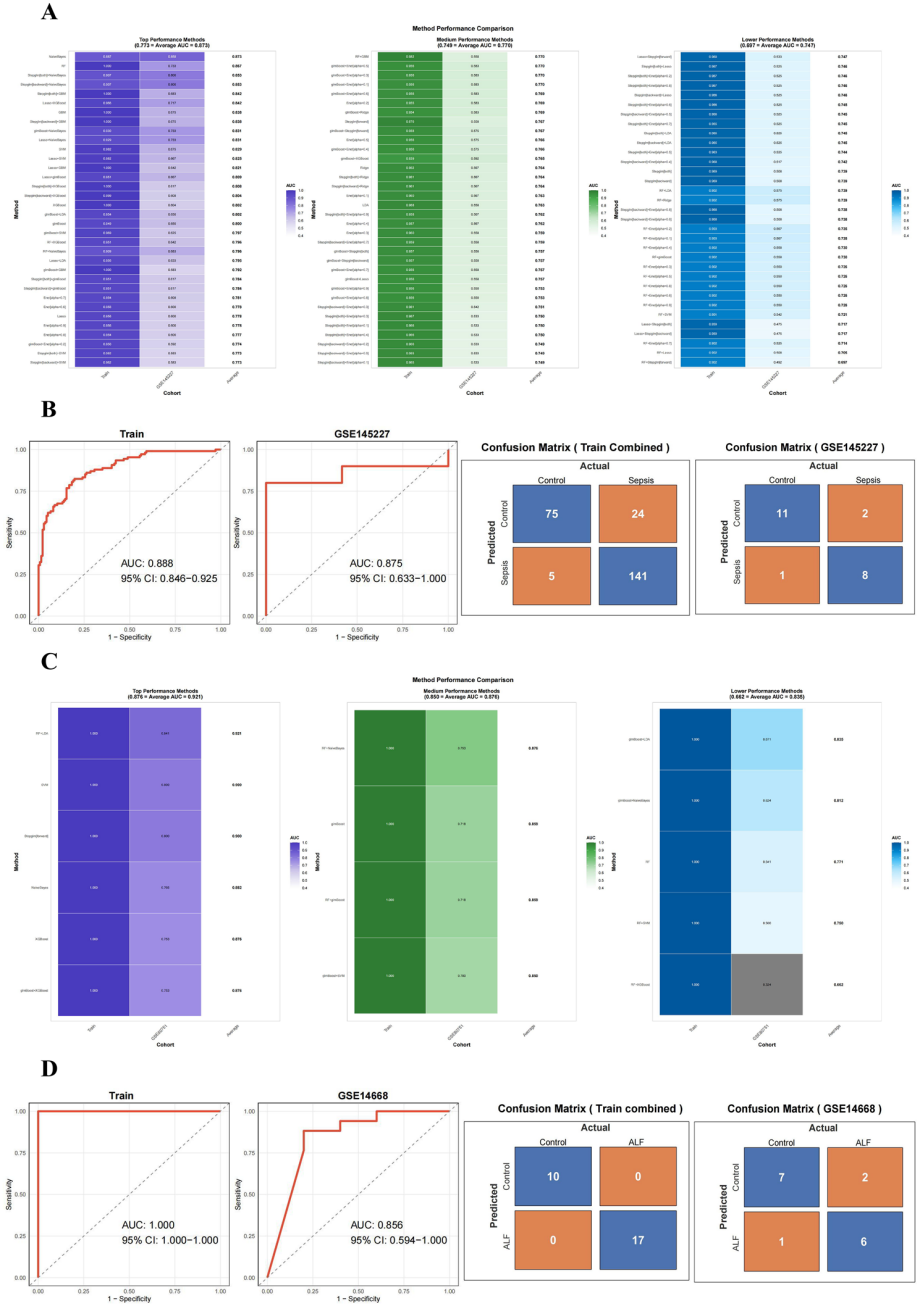
Machine learning results analysis. (**A**) Results of machine learning algorithms by sepsis; (**B**) ROC curves and confusion matrix of “NaiveBayes” algorithm by sepsis; (**C**) Results of machine learning algorithms by ALF; (**D**) Results of “ROC” algorithm by ALF

Based on 10-fold cross-validation, a total of 12 machine learning algorithms were combined to determine the optimal diagnostic model for the 40 intersecting genes. Figure 4A shows the AUC values of sepsis models using different algorithm combinations. Among them, the algorithm with the highest average AUC value, “Naive Bayes,” identified 39 genes, as detailed in Supplementary Table S5A. In addition, the algorithms with higher average AUC values also include “RF,” “Stepglm[both] + Naive Bayes,” “Stepglm[backward] + Naive Bayes,” “Stepglm[both] + GBM,” and “Lasso + XGBoost.” In order to further strengthen the stability and reliability of the diagnostic model, as well as to comprehensively evaluate the model’s performance, the aforementioned machine learning algorithms were used for further analysis. We eventually found that the ROC and confusion matrix results using the “Naive Bayes” algorithm were superior. The ROC curve shows that the AUC value of the “Naive Bayes” algorithm model gene in the training set is 0.888 (95% CI: 0.846–0.925), and in the test set (GSE145227) the AUC value is 0.875 (95% CI: 0.633-1.000), both indicating good predictive levels (Fig. 4B). The confusion matrix shows that the “Naive Bayes” algorithm has an accuracy of 0.859, precision of 0.852, recall of 0.965, and specificity of 0.756 in the training set; and an accuracy of 0.846, precision of 0.800, recall of 0.889, and specificity of 0.846 in the test set, indicating that the algorithm has good predictive performance (Fig. 4B). These results reveal a high consistency between the predictions generated by the sepsis prediction model and the actual clinical outcomes, confirming the reliability of the model’s calibration performance.

In ALF, the algorithm with the highest average AUC, “RF + LDA,” identified 18 genes (Fig. 4C, Supplementary Table S5B). The algorithms with higher average AUC values include “SVM,” “Stepglm[forward],” “NaiveBayes,” “XGBoost,” and “glmBoost + XGBoost.” Again, we plotted the ROC and confusion matrix for these algorithms, and we found that the “RF + LDA” algorithm had better predictive power. The ROC curve results indicate that the AUC of the “RF + LDA” algorithm model genes in the training set is 1.000 (95% CI: 1.000–1.000) and 0.856 (95% CI: 0.594-1.000) in the test set (GSE14668) (Fig. 4D). The confusion matrix results show that the “RF + LDA” algorithm model has an Accuracy of 0.941, Precision of 1.000, Recall of 1.000, and Specificity of 1.000 in the training set; in the test set, it has an Accuracy of 0.750, Precision of 0.750, Recall of 0.857, and Specificity of 0.778, all indicating good predictive performance (Fig. 4D).

As we all know, the “RF” and “LDA” algorithms are more suitable for complex or high-dimensional datasets, which may be a significant reason why the “RF + LDA” algorithm cannot fully leverage its advantages in the simpler ALF data. On the other hand, the “Naive Bayes” algorithm performs better on datasets with strong feature independence, which we speculate may be related to different data characteristics. In summary, we ultimately chose the sepsis model genes from the “Naive Bayes” algorithm and the ALF model genes from the “RF + LDA” algorithm.

### Evaluation of feature genes

**Fig. 5:**
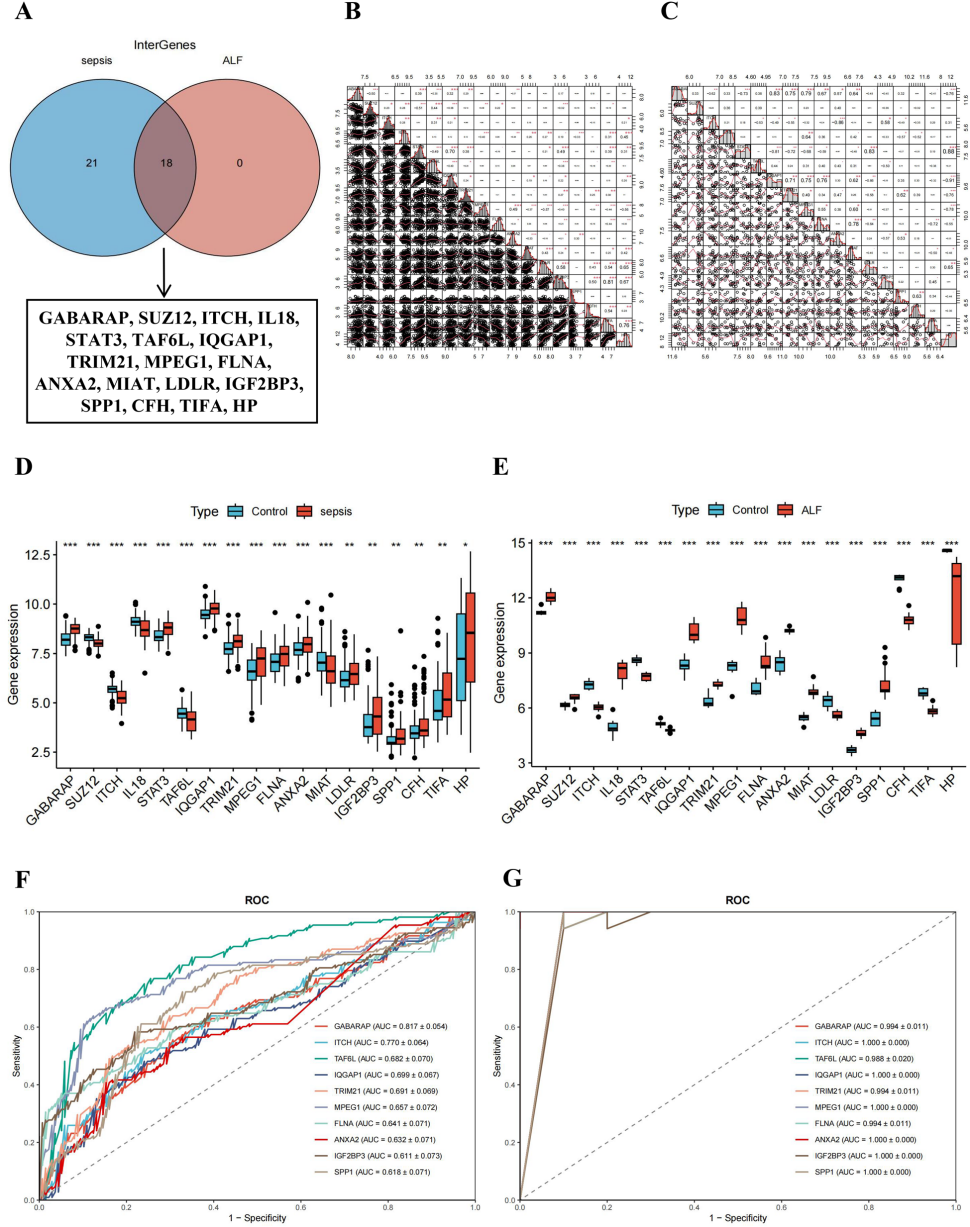
Evaluation of feature genes. (**A**) Venn diagram of the intersection of sepsis model genes and ALF model genes; (**B**) Correlation plot of intersected genes in sepsis; (**C**) Correlation plot of intersected genes in ALF; (**D**) Box plot of intersected genes in sepsis; (E) Box plot of intersected genes in ALF; (**F**) ROC of intersected genes in sepsis; (**G**) ROC of intersected genes in ALF

The intersection of the sepsis model genes from the “NaiveBayes” algorithm and the ALF model genes from the “RF + LDA” method generated 18 shared genes (Fig. 5A). The association among these 18 shared genes in sepsis and ALF were illustrated individually (Fig. 5B-C). Box plots indicated that GABARAP, IQGAP1, TRIM21, MPEG1, FLNA, ANXA2, IGF2BP3, and SPP1 were upregulated genes in both sepsis and ALF, while ITCH and TAF6L were downregulated genes in both conditions (Fig. 5D-E). The ROC curves of these 10 model genes that were consistently upregulated or downregulated in sepsis and ALF were created, revealing that the AUC values of the two critical genes, GABARAP and ITCH, were ≥ 0.7 in both conditions (Fig. 5F-G).The results indicated that GABARAP and ITCH might have played significant roles in influencing the onset and progression of sepsis combined with ALF.

### Hub genes enrichment analysis

**Fig. 6:**
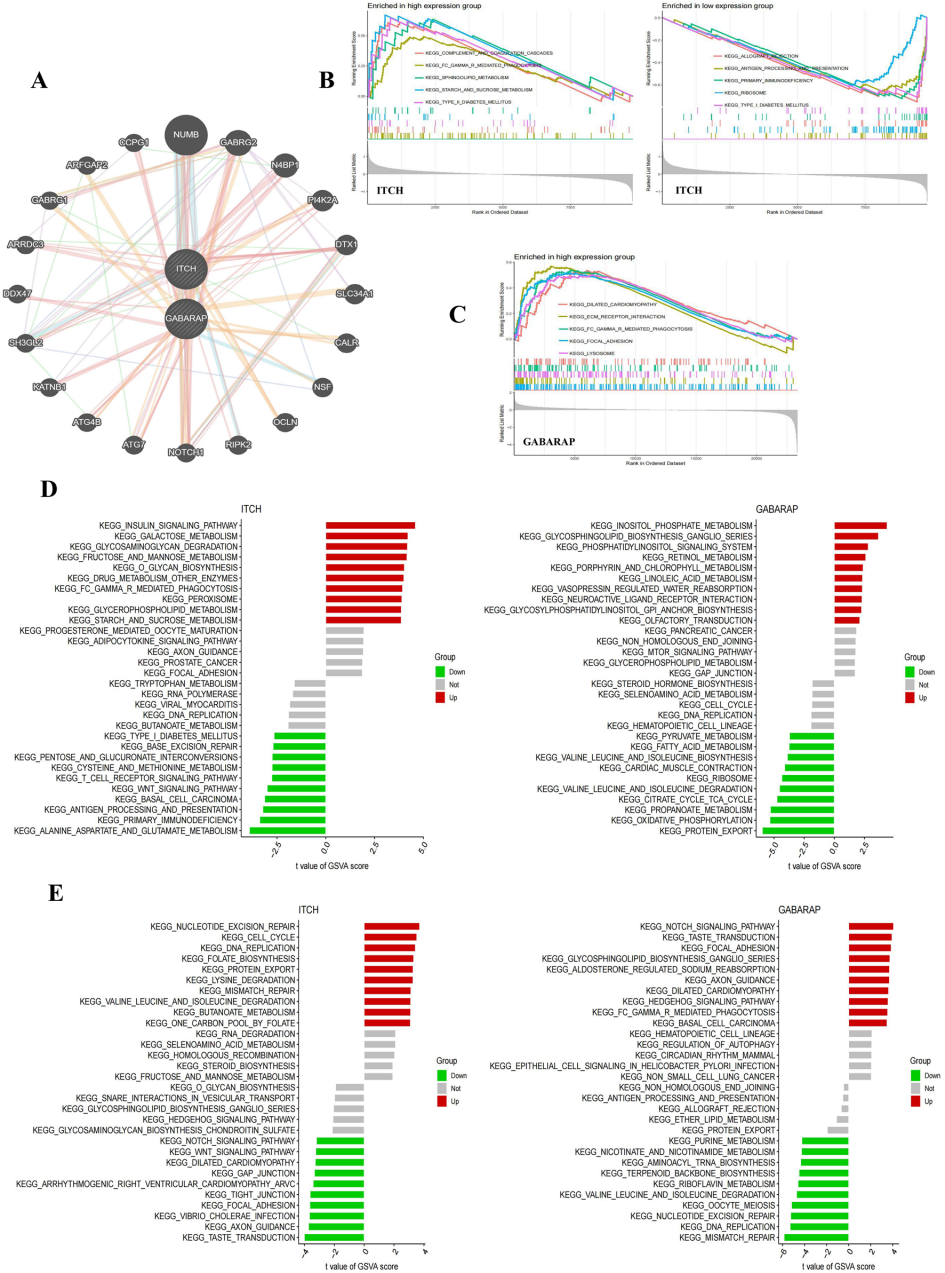
PPI network analysis and enrichment analyses. (**A**) PPI network of the 2 hub genes constructed by GeneMANIA; (**B**) GSEA of ITCH in sepsis databases; (**C**) GSEA of GABARAP in ALF databases; (**D**) GSVA of ITCH and GABARAP in sepsis databases; (E) GSVA of ITCH and GABARAP in ALF databases

The PPI network showed the connection network between GABARAP and ITCH8, comprising two feature genes, 20 associated genes, and 132 edges (Fig. 6A). The GSEA results indicated ITCH was predominantly implicated in primary immunodeficiency, FcγR-mediated phagocytosis, and the T cell receptor signaling pathway inside sepsis dataset. (Fig. 6B, Supplementary Table S6), while GABARAP showed a strong correlation with FcγR-mediated phagocytosis, T cell receptor signaling route, B cell receptor signaling pathway in the ALF dataset (Fig. 6C, Supplementary Table S6). The GSVA results indicated that, in both two conditions datasets, GABARAP participated in protein export, degradation of valine, leucine, and isoleucine, non-homologous end joining, and ganglioside biosynthesis, while ITCH primarily engaged in butyric acid metabolism and fructose and mannose metabolism (Fig. 6D-E, Supplementary table S7).

Additionally, the GSEA results indicated that both GABARAP and ITCH are involved in the T cell receptor signaling pathway, and ITCH is implicated in the B cell receptor signaling pathway in the ALF dataset. The results suggest the two identified genes may influence the pathogenic mechanisms of sepsis and ALF by modulating the body’s immune response.

### Examination of immune cell infiltration

**Fig. 7:**
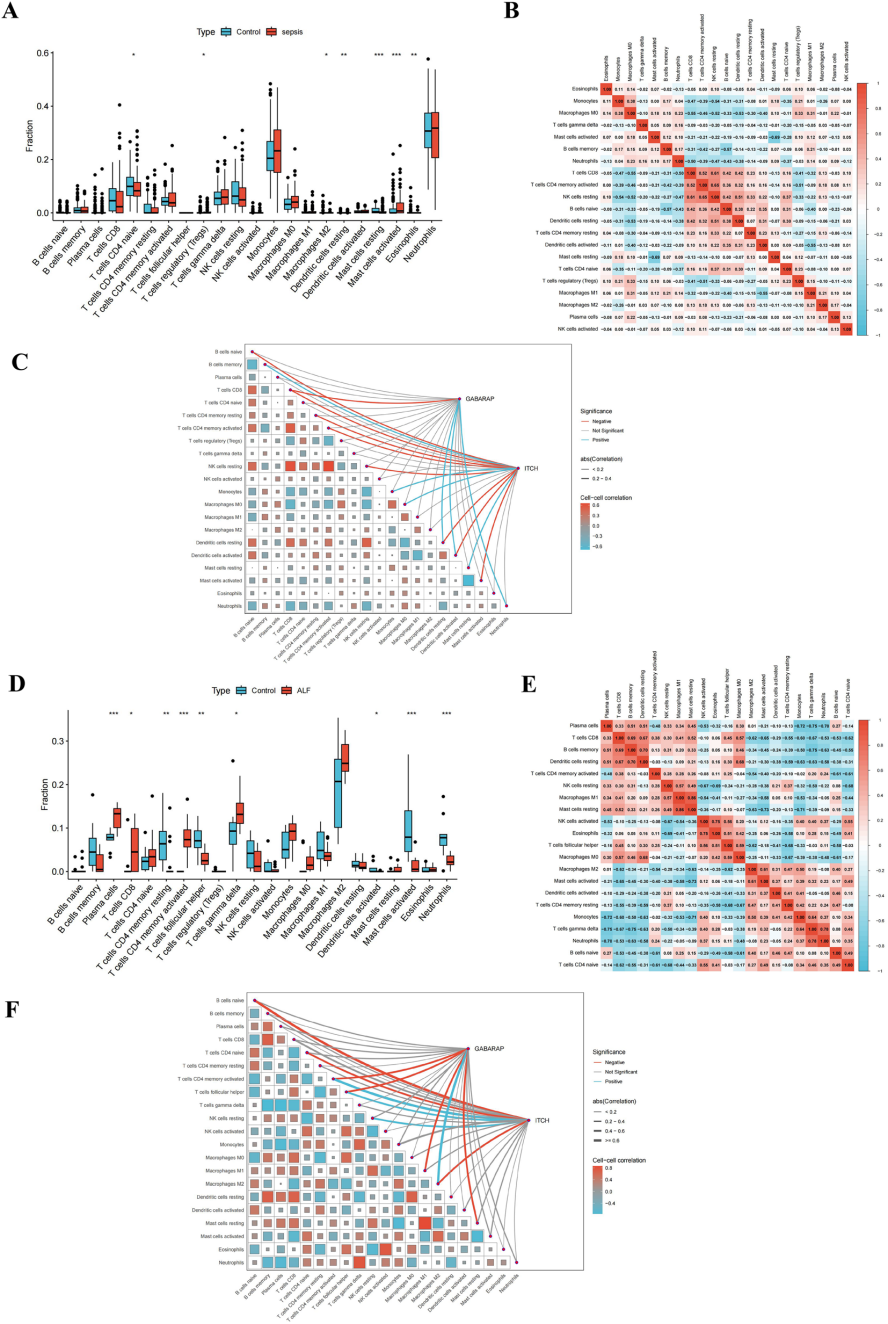
Immune cell infiltration analysis. (**A**) sepsis immune cell infiltration box plot; (**B**) heatmap of immune cell infiltration in sepsis; (**C**) sepsis immune cell correlation analysis with the expression of 2 hub genes; (**D**) ALF immune cell infiltration box plot; (**E**) heatmap of immune cell infiltration in ALF; (**F**) ALF immune cell correlation analysis with the expression of 2 hub genes. *indicates significance level (*P < 0.05, **P < 0.01, ***P < 0.001)

The sepsis dataset revealed a substantial elevation in the level of activated mast cells, while the levels of naive CD4 + T cells and resting mast cells were dramatically diminished (Fig. 7A). Activated mast cells demonstrated an inverse correlation with resting mast cells; naive CD4 + T cells revealed a direct link with resting NK cells (Fig. 7B). GABARAP was negatively correlated with CD8 + T cells and positively correlated with resting dendritic cells, activated dendritic cells, and neutrophils. ITCH showed a negative correlation with naive B cells, CD8 + T cells, resting CD4 + memory T cells, and resting CD4 + T cells, while positively correlating with memory B cells, monocytes, M0 macrophages, and mast cells resting (Fig. 7C).

In the ALF dataset, the levels of plasma cells, CD8 + T cells, CD4 + T cells memory activated, and gamma delta T cells were significantly elevated, while the levels of CD4 + T cells memory resting, follicular helper T cells, CD8 + T cells activated and neutrophils were dramatically decreased (Fig. 7D). Plasma cells showed a negative correlation with gamma delta T cells. CD8 + T cells exhibited a positive correlation with memory B cells. Activated CD4 + memory T cells had a negative correlation with naive B cells and naive CD4 + T cells. Gamma delta T cells have a positive correlation with neutrophils. Resting CD4 + memory T cells had a negative correlation with follicular helper T cells. Activated dendritic cells had a positive correlation with naive B cells. Activated mast cells had a negative correlation with resting mast cells (Fig. 7E). Figure 7F illustrates that GABARAP had a negative correlation with activated CD4 + memory T cells, follicular helper T cells, M1 macrophages and resting mast cells, while demonstrating a positive correlation with M2 macrophages. ITCH had a negative correlation with naive B cells, naive CD4 + T cells, and M2 macrophages, while showing a positive correlation with activated CD4 + memory T cells, follicular helper T cells, and resting NK cells.

These findings indicate that these two hub genes may significantly influence the cellular immune response, particularly via T cells, in the critical pathophysiology of sepsis combined with ALF.

### Animal experiment results

**Fig. 8:**
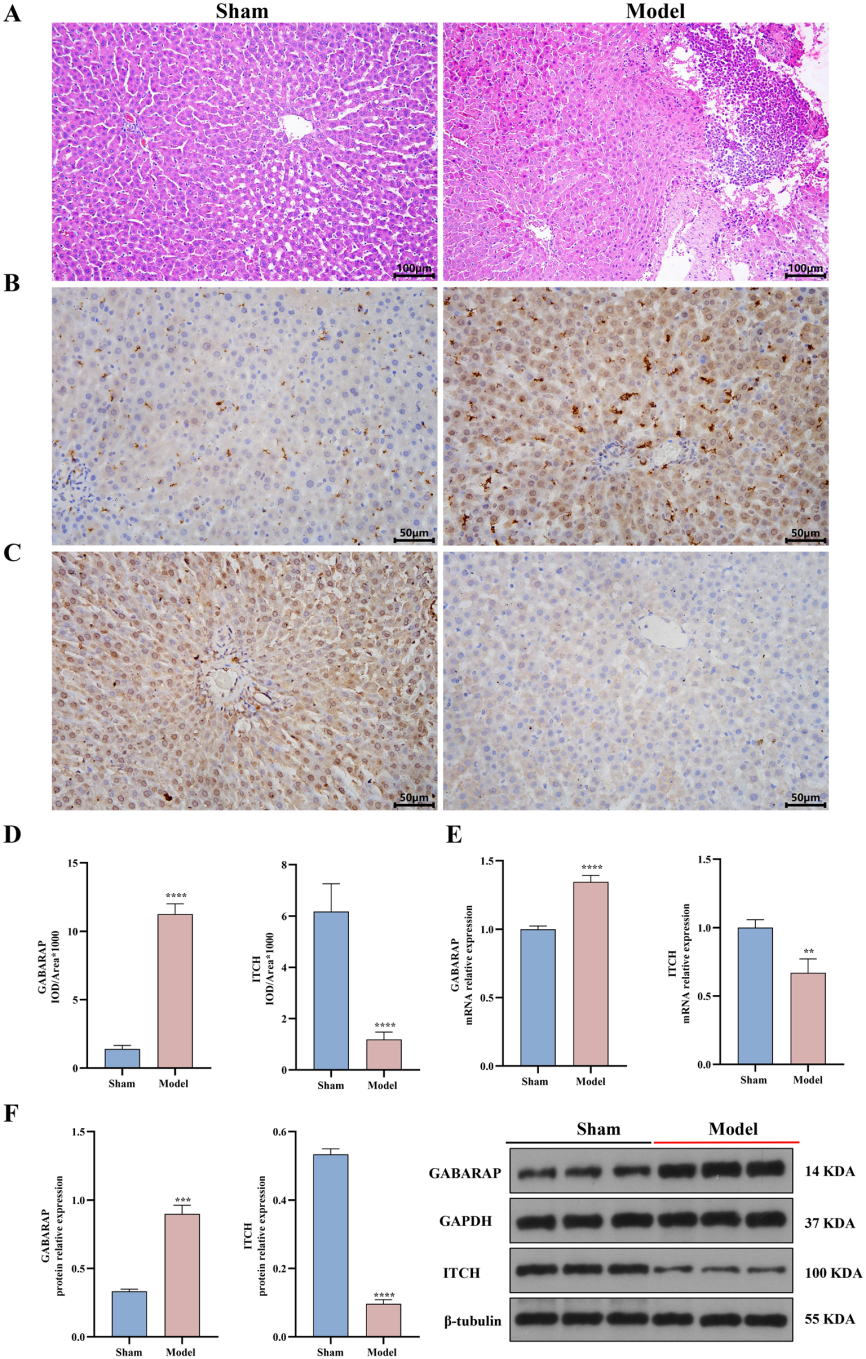
Validation results of animal experiments. **A**. HE staining to observe the histopathological changes of liver in sham and model groups; **B**.Immunohistochemistry to detect the expression level of GABARAP in the two groups; **C**. Immunohistochemistry to detect the expression level of ITCH in the two groups; **D**. Histogram to show the expression level of GABARAP and ITCH in the two groups (Immunohistochemistry); **E**. qRT-PCR to detect the expression level of GABARAP and ITCH mRNA in the two groups; **F**. Western blot to detect the expression level of GABARAP and ITCH in the two groups. *indicates significance level (compared with the sham group, *P < 0.05, **P < 0.01, ***P < 0.005, ****P < 0.001)

HE staining indicated obvious liver histopathological damage in the sepsis combined with ALF model group, characterized by marked structural disarray of liver lobules, disorganized hepatocyte arrangement, significant inflammatory cell infiltration, and hepatocyte degeneration (Fig. 8A). Immunohistochemistry indicated that the expression level of GABARAP in the model group was significantly elevated (*P* < 0.001) (Fig. 8B and D), while the expression level of ITCH was dramatically diminished (*P* < 0.001) (Fig. 8C and D). qRT-PCR results indicated (Fig. 8E) that, compared to the sham-operated group, the GABARAP mRNA expression in the model group was significantly elevated (*P* < 0.001). In contrast, the ITCH mRNA expression level drastically decreased (*P* < 0.01). The Western blot analysis indicated that, compared to the sham-operated group, the GABARAP protein expression level was significantly elevated (*P* < 0.001). In contrast, the ITCH protein expression level was dramatically diminished (*P* < 0.001) in the model group (Fig. 8F).

## Discussion

Pyroptosis is a type of programmed cell death characterized by inflammation, which efficiently preserves the host’s protection against pathogen infection [12]. Research has shown that pyroptosis may significantly contribute to the onset and progression of sepsis and ALF [14, 16]. Moreover, pyroptosis may proficiently facilitate the immunological response by stimulating CD8 + T cells and NK cells, as well as modulating the metabolism and development of neutrophils and other immune-related cells [19, 20]. This study investigated the shared diagnostic genes of sepsis and ALF from the perspective of pyroptosis, to clarify the interplay between pyroptosis and immune response, as well as its biological mechanisms, thereby offering a fresh strategy and theoretical foundation for the prevention and management of sepsis combined with ALF.

Machine learning indicated that GABARAP and ITCH may be hub genes implicated in the pathological mechanisms of sepsis and ALF. Bioinformatics analyses demonstrated their significant correlation with FcγR-mediated phagocytosis, T cell receptor signaling pathways, B cell receptor signaling pathways, and other closely related biological processes. The findings regarding immune cell infiltration indicated that in sepsis, there was an elevation in mast cells activated infiltration, while infiltration of naive CD4 + T cells and resting mast cells diminished; in ALF, there was an elevation in plasma cells, CD8 + T cells, activated CD4 + memory T cells, and gamma delta infiltration T cells; conversely, infiltration of resting CD4 + memory T cells, follicular helper T cells, activated dendritic cells, activated mast cells and neutrophils diminished. The analysis of immune cell infiltration indicated that immune dysregulation is present in both sepsis and ALF. Considering the intricate relationship between immune cells and sepsis as well as ALF, the correlation of GABARAP and ITCH with various immune cells was analyzed. The results revealed that these two hub genes exhibited varying degrees of both positive and negative correlations with a range of immune cells, including resting mast cells, activated CD4 + memory T cells, and NK cells resting. Moreover, animal experimental investigations supported the validity of the findings. Additionally, further animal experiments have indicated that GABARAP and ITCH may mediate the pathogenesis of sepsis combined with ALF. In conclusion, GABARAP and ITCH may serve as promising pyroptosis-related diagnostic biomarkers for sepsis and ALF.

γ-Aminobutyric acid A receptor-associated protein (GABARAP) was first identified by yeast two-hybrid screening for proteins that interact with the GABAA receptor [46]. GABARAP has a critical function in regulating inflammatory reactions and cellular pyroptosis. GABARAP deficiency impairs mitochondrial homeostasis in macrophages and enhances mitochondrial ROS generation, hence exacerbating NLRP3 inflammasome-dependent inflammatory responses and contributing to critical pathogenic processes in sepsis [47]. This study revealed that GABARAP expression was increased in a diagnostic model of sepsis combined with ALF. Our research used human tissue or serum samples from the GEO database and was corroborated with an external dataset, supporting the credibility of our findings. In addition, there is a paucity of research regarding the correlation between GABARAP and ALF, with barely any research validating the role of GABARAP in sepsis combined with ALF. Therefore, it is imperative to further investigate the relationship between this gene and sepsis combined with ALF.

Itchy E3 Ubiquitin Protein Ligase Homolog (ITCH) is an E3 ubiquitin ligase of the HECT family, significantly participating in ubiquitination modification and degradation of various cellular proteins and effectively regulating immune responses and inflammation in the organism [48]. The expression of CircRNA ITCH (circ-ITCH) is associated with ITCH, and circ-ITCH may influence ITCH expression through regulating miRNAs [48]. Prior research has showed that the overexpression of circ-ITCH effectively ameliorates mitochondrial dysfunction, hence providing a therapeutic benefit for sepsis-related acute kidney damage [49]. Another study’s results indicated that the knockout of ITCH significantly suppressed the activation of NLRP1 inflammatory vesicles, therefore mitigating the immune-inflammatory response [50]. Also, it has been proposed that genetic alterations in ITCH may result in ALF [51]. This study revealed a reduced expression level of ITCH in the diagnostic model of sepsis combined with ALF. But no research has yet substantiated the involvement of ITCH in the pathophysiology of sepsis combined with ALF, requiring further investigation.

GABARAP and ITCH may have extensive regulatory effects on numerous biological pathways, particularly concerning cellular immune function, which could impact the pathogenesis of sepsis combined with ALF. GABARAP has been demonstrated to target antigen-sensitized dendritic cells and activate CD4 + T cells and CD8 + T cells, thereby initiating an organismal immune response [52], while the absence of ITCH in mice enhances T cell activation, aggravating autoimmune responses [53]. Moreover, abundant prior evidence supports immunological dysregulation being a fundamental pathological mechanism in sepsis and ALF [54–57]. Septic mice exhibit an exhaustion in CD4 + T cells, resulting impairment in compromised adaptive immune responses [54]; patients and mice with ALF show infiltration of CD8 + T cells in hepatic tissues, which stimulates the immune system and worsens liver damage [56]. These findings suggest that GABARAP and ITCH may facilitate immunological dysregulation, thereby contributing to the development of sepsis and ALF.

The findings on immune cell infiltration suggest that the malfunction of cellular immunity, resulting from the interaction of various immune cells, may be the principal pathogenic mechanism underlying sepsis combined with ALF. The modulation of immune receptors on mast cells and dendritic cells effectively facilitated neutrophil recruitment in septic mice [58]; modulation of CD4 + T cell migration and cytokine secretion significantly intervened acute liver injury in mice [59]; exhaustion of CD8 + T cells reduced acute liver injury in mice [56]; and sepsis was primarily characterized by an altered CD8 + T cell composition, which directly influences the CD8 + T cell response to the initial infection [60]. Overall, the occurrence and development of sepsis and ALF may be related to the role of multiple immune cells.

This study possesses multiple strengths: (i) By utilizing various bioinformatics analytical tools and machine learning methodologies, we have provided a novel and in-depth examination of the biological basis of sepsis and ALF, identifying two hub genes, GABARAP and ITCH, which may serve as potential diagnostic markers related to pyroptosis in these diseases. (ii) We have revealed the potential correlation between these hub genes and the cellular immune response, offering valuable insights for a more profound comprehension of the disease mechanism and suggesting potential directions for future research. (iii) This study provides new concepts and methodologies for investigating therapeutic solutions for sepsis with ALF, laying the groundwork for further research. In summary, this study enhances the comprehension of the molecular mechanisms underlying sepsis combined with ALF. It opens up new avenues for research and therapeutic strategies to address this comorbidity, thereby advancing the field of critical care medicine.

Our study identified two hub genes associated with pyroptosis, which may play a critical part in the pathogenic mechanisms of sepsis and ALF. However, several limits exist: (i) The present sample size for sepsis and ALF is limited, potentially compromising the accuracy of the findings. Subsequent research requires more extensive datasets to validate the findings. (ii) While possible hub genes have been identified and validated by basic animal tests, further comprehensive in vitro, in vivo, and clinical investigations are required to clarify the specific link between these genes and sepsis associated with ALF. (iii) This analysis merely utilized the GEO-database sepsis and ALF datasets, failing to sufficiently account for confounding variables such as nutrition, schedule, age, and gender, which may introduce bias. Future studies should more effectively regulate and evaluate these aspects to achieve more precise and reliable results. (iv) The pathophysiology of sepsis and ALF is complicated, involving the interplay of genetic, environmental, and other molecular processes. This study concentrates on particular pyroptosis-related genes but may not include all molecular pathways involved in the pathophysiology of sepsis and ALF. Further pharmacological investigations are required to confirm the therapeutic efficacy of targeting these genes for sepsis combined with ALF.

## Conclusion

This study reveals the potential value of GABARAP and ITCH as diagnostic biomarkers associated with pyroptosis in sepsis and ALF. These diagnostic biomarkers may significantly contribute to the onset and progression of sepsis combined with ALF via immunomodulatory processes. Furthermore, we validated the significance of these diagnostic biomarkers through animal experiments and proposed novel strategies for the treatment of sepsis combined with ALF. These findings may provide the groundwork for the long-term diagnostic strategies, prognostic indicators, and treatment targets for sepsis combined with ALF.

## Supplementary Information

Below is the link to the electronic supplementary material.

Supplementary Material 1

Supplementary Material 2

Supplementary Material 3

## Data Availability

Data is provided within the manuscript or supplementary information files.
