# Supplementary material for: Integrating bioinformatics analysis, machine learning, and experimental validation to identify pyroptosis-related genes in the diagnosis of sepsis combined with acute liver failure
Source: Hereditas. 2025 Aug 8;162:153. doi: 10.1186/s41065-025-00522-4 (PMC12333193; doi:10.1186/s41065-025-00522-4)
Supplement: Supplementary file 2 — Supplementary Material 2 [file 41065_2025_522_MOESM2_ESM.docx]

Introduction to 12 Machine Learning Algorithms

1. **Lasso**

The basic idea of LASSO (Least Absolute Shrinkage and Selection Operator) is to minimize the sum of squared residuals under the constraint that the sum of the absolute values of the regression coefficients is less than a constant. This can produce some regression coefficients that are strictly equal to 0, resulting in an interpretable model. The mathematical expression is as follows:


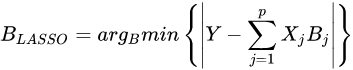


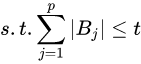


Where *t* > 0 is a tuning parameter. By controlling the tuning parameter t, the overall regression coefficients can be compressed. The value of t can be determined using the cross-validation method proposed by Efron and Tibshirani (1993). This mathematical expression is also equivalent to minimizing the following penalized least squares:


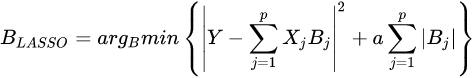


Where *a* and *t* correspond to each other and can be mutually converted. The main advantages of the LASSO method lie in its ability to compress variables with larger parameter estimates to a lesser degree, while variables with smaller parameter estimates are compressed to zero. Moreover, LASSO analysis provides continuous parameter estimates, making it suitable for model selection in high-dimensional data. In 2005, Tibshirani proposed the Fused LASSO method, an estimation approach that satisfies the sparsity of model coefficients as well as the difference between coefficients, making adjacent coefficients smoother.

1. **Ridge**

The Ridge regression algorithm is a regularization method used in linear regression to handle cases where there is collinearity (i.e., high correlation) among features. It was proposed by Tikhonov in the 1960s, and is also known as Tikhonov regularization. Ridge regression constrains the model's parameters by introducing an L2 regularization term into the loss function. Its optimization objective is to find a model that can both fit the training data and have relatively small parameters. By introducing the regularization term, Ridge regression forces the model to minimize the values of the parameters, thus preventing overfitting. The introduction of the regularization term restricts the model's parameters, making them smoother and more stable.

1. **Elastic Net Regression**

The cost function of the Elastic Net regression algorithm combines the regularization methods of Lasso regression and Ridge regression, using two parameters λ and ρ to control the size of the penalty term.


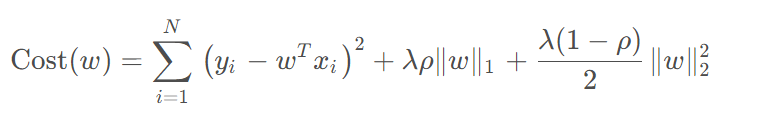


Similarly, it seeks to find the size of w that minimizes the cost function:


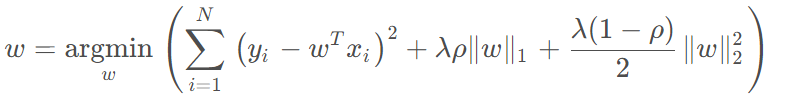


It can be seen that when ρ = 0, its cost function is equivalent to the cost function of Ridge regression. When ρ = 1, its cost function is equivalent to the cost function of Lasso regression. Like Lasso regression, there are absolute values in the cost function, which are not derivable everywhere, so it is not possible to directly obtain the analytical solution of w by directly deriving. However, we can still use the coordinate descent method to solve for w.

1. **Stepglm**

Stepglm is a stepwise regression method commonly used for variable selection in generalized linear models (GLM). Specifies the direction of the stepwise regression, with options including "forward" (forward selection), "backward" (backward elimination), and "both" (bidirectional selection).Defines the range of changes for the model formula, used to restrict or specify the variables available for selection in the stepwise regression. For example, scope = list(lower = formula1, upper = formula2), where formula1 and formula2 respectively denote the lower and upper bounds of the model formula.

A criterion used to determine whether a variable enters or is removed from the model. It is usually set to 2 and is related to the Akaike Information Criterion (AIC). When considering the Bayesian Information Criterion (BIC), set k = log(n), where n is the sample size.Sets the maximum number of iterations for the stepwise regression, with a default value typically of 1000. Controls whether detailed information during the stepwise regression process is displayed. By default, it is TRUE, which shows detailed information at each step, including variable entry or removal and changes in corresponding statistics. If set to FALSE, only the final result is displayed.Specifies the error distribution family and the link function, defining the response distribution and the association function of the model. For instance, binomial(link = 'logit') indicates a binomial distribution with the logit link function, suitable for classification problems.Defines how missing values are handled, such as na.omit, which directly removes observations with missing values.Used to set the variance estimate of the error term when using certain distribution families (e.g., Gaussian distribution).An initial model can be provided, and the stepwise regression will perform variable addition or removal based on this model.

1. **SVM**

Support Vector Machine (SVM), also known as Support Vector Network, is a supervised learning model and associated learning algorithms used for classification and regression analysis. Given a set of training samples, each training sample is labeled as belonging to one of two categories. The SVM training algorithm creates a model that assigns new samples to one of the two categories, making it a non-probabilistic binary linear classifier (although there are methods like Platt scaling that use Support Vector Machine in probability classification settings). The SVM model represents samples as points in space, such that samples of a single category are separated by a clear gap as much as possible. When all new samples are mapped to the same space, they can be predicted to belong to a category based on which side of the gap they fall on.

1. **glmBoost**

GlmBoost (Gradient Boosted Generalized Linear Models) algorithm is a machine learning algorithm that combines gradient boosting and generalized linear models (Glms). It is widely used for regression and classification problems. Gradient boosting is an ensemble learning technique that sequentially trains multiple weak prediction models (usually decision trees), each of which tries to correct the errors made by the previous models. The weak models are combined together to form a powerful prediction model. On the other hand, generalized linear models are a class of models that generalize linear regression to other types of response variables. Glms are suitable for cases where the response variable follows a specific distribution, such as the binomial distribution for binary classification or the Poisson distribution for count data. GlmBoost combines these two techniques by fitting a series of Glms to the residuals generated during the gradient boosting process. Each Glm in the series is trained to minimize a specific loss function, usually deviance. The final prediction result is obtained by combining the predictions from all Glms in the series. The algorithm iteratively fits Glms to the negative gradient of the loss function, thereby optimizing for the specific problem. The learning rate plays an important role in the training process, controlling the contribution of each individual model. GlmBoost has several advantages over other algorithms. It can handle various types of response variables, including binary, count, and continuous variables. It is robust to outliers and noise in the data. Moreover, Glms themselves are more interpretable than other machine learning algorithms, such as neural networks. Overall, GlmBoost is a powerful algorithm that combines the advantages of gradient boosting and generalized linear models. It has been successfully applied to various fields, including healthcare, finance, and marketing, to solve regression and classification tasks.

1. **LDA**

LLinear Discriminant Analysis (LDA) belongs to the category of supervised learning algorithms in machine learning, commonly used for feature extraction, data dimensionality reduction, and task classification. It plays an essential role in fields such as face recognition and face detection. LDA algorithm and PCA (Principal Component Analysis) algorithm are both commonly used dimensionality reduction techniques. The difference between the two lies in the fact that LDA is a supervised learning dimensionality reduction technique, meaning each sample has a category output, whereas the PCA algorithm we learned before is an unsupervised dimensionality reduction technique that does not consider the category output of samples. The goal of the LDA algorithm is to minimize the within-class variance and maximize the between-class variance after dimensionality reduction (i.e., it aims to have the projected points of each category data as close as possible while maximizing the distance between the centers of different category data in the low-dimensional space after projection).

1. **plsRglm**

plsRglm is an algorithm based on partial least squares (PLS) and generalized linear models (GLMs). It is used to handle situations with multiple predictor variables and one response variable and is generally applicable to regression and classification problems. Partial Least Squares (PLS) is a multivariate regression technique that maximizes predictive ability by transforming predictor variables and response variables and establishing a linear relationship in the transformed space. Compared to other regression methods, PLS can handle highly correlated predictor variables and provide more reliable predictive results. Generalized linear models are a class of models that generalize linear regression to other types of response variables. Unlike ordinary linear regression, GLMs can choose the appropriate link function and loss function according to the distribution characteristics of the response variable, such as binomial distribution, Poisson distribution, etc. plsRglm combines the concepts of PLS and GLMs, offering a powerful modeling approach. It uses principal component analysis to reduce predictor variables and then fits a generalized linear model to the remaining principal components. The algorithm iteratively performs this process until the predetermined number of iterations or convergence is reached. The plsRglm algorithm can solve high-dimensional data modeling problems with fewer predictor variables. Moreover, omitting redundant information makes the model easier to interpret and understand.

In conclusion, the plsRglm algorithm combines the advantages of partial least squares and generalized linear models, holding significant application value in multivariate modeling and analysis. It is applicable to various fields, such as chemistry, biology, and finance, effectively handling regression and classification problems.

1. **Random Forest**

Random Forest is an ensemble learning algorithm used for regression and classification problems. It consists of a collection of decision trees, and the final prediction result is obtained by combining the predictions of each tree. The key idea of Random Forest is to construct each decision tree by randomly selecting subsets of features and samples. This randomness helps to avoid overfitting and increases the model's stability and accuracy. Random Forest is suitable for large-scale data and problems with many feature dimensions. It has the advantages of being less prone to overfitting and having lower variance. Random Forest is widely used in various fields, including financial risk assessment, medical diagnosis, image classification, and natural language processing. It is a powerful and flexible machine learning algorithm often regarded as a potent tool for solving complex problems.

1. **GBM**

The Gradient Boosting Machine (GBM) algorithm is a machine learning algorithm based on decision trees. It gradually improves predictive performance by continuously fitting the residuals of errors. GBM is an ensemble learning algorithm that can integrate multiple weak classifiers to form a strong classifier. During the training process, GBM improves the accuracy of the classifier through multiple iterations, eventually obtaining a strong classifier with high accuracy.

1. **XGBoost**

XGBoost (eXtreme Gradient Boosting) is an ensemble learning algorithm based on Gradient Boosting Decision Trees. Compared to traditional GBM (Gradient Boosting Machine), XG Boost has optimized and improved performance and scalability. It has shown excellent performance in various machine learning tasks and is particularly suitable for regression and classification problems. XGBoost has achieved widespread success in multiple machine learning competitions and practical applications. It has been widely used in fields such as finance, e-commerce, recommendation systems, and search ranking, and has become an important algorithm in the toolboxes of many data science practitioners.

1. **Naive Bayes**

The NB (Naive Bayes) algorithm, also known as the Naive Bayesian algorithm, is a classic and representative classification algorithm. This algorithm is a classification method based on Bayes' theorem and is derived using the posterior probability formula. In the NB algorithm, the "attribute conditional independence assumption" is adopted: assuming that all attributes are independent of each other given a known category. In other words, it is assumed that each attribute independently affects the classification result. The NB algorithm can handle both symbolic data and numerical data. The "naive" in Naive Bayes represents the assumption that features are independent of each other. However, in reality, many attributes are not independent of each other. Therefore, this is an ideal state. The NB algorithm is a probability-based classification algorithm, and therefore, a significant portion of this article is devoted to formula derivation. The Naive Bayes algorithm is easy to understand and implement and is commonly used in text classification, spam email classification, recommendation systems, and more.
