# Supplementary material for: Integrating bioinformatics analysis, machine learning, and experimental validation to identify pyroptosis-related genes in the diagnosis of sepsis combined with acute liver failure
Source: Hereditas. 2025 Aug 8;162:153. doi: 10.1186/s41065-025-00522-4 (PMC12333193; doi:10.1186/s41065-025-00522-4)

1、GABARAP（A12568, Abclonal, 1/1000）；GAPDH (T0004, Affinity, 1/10000)


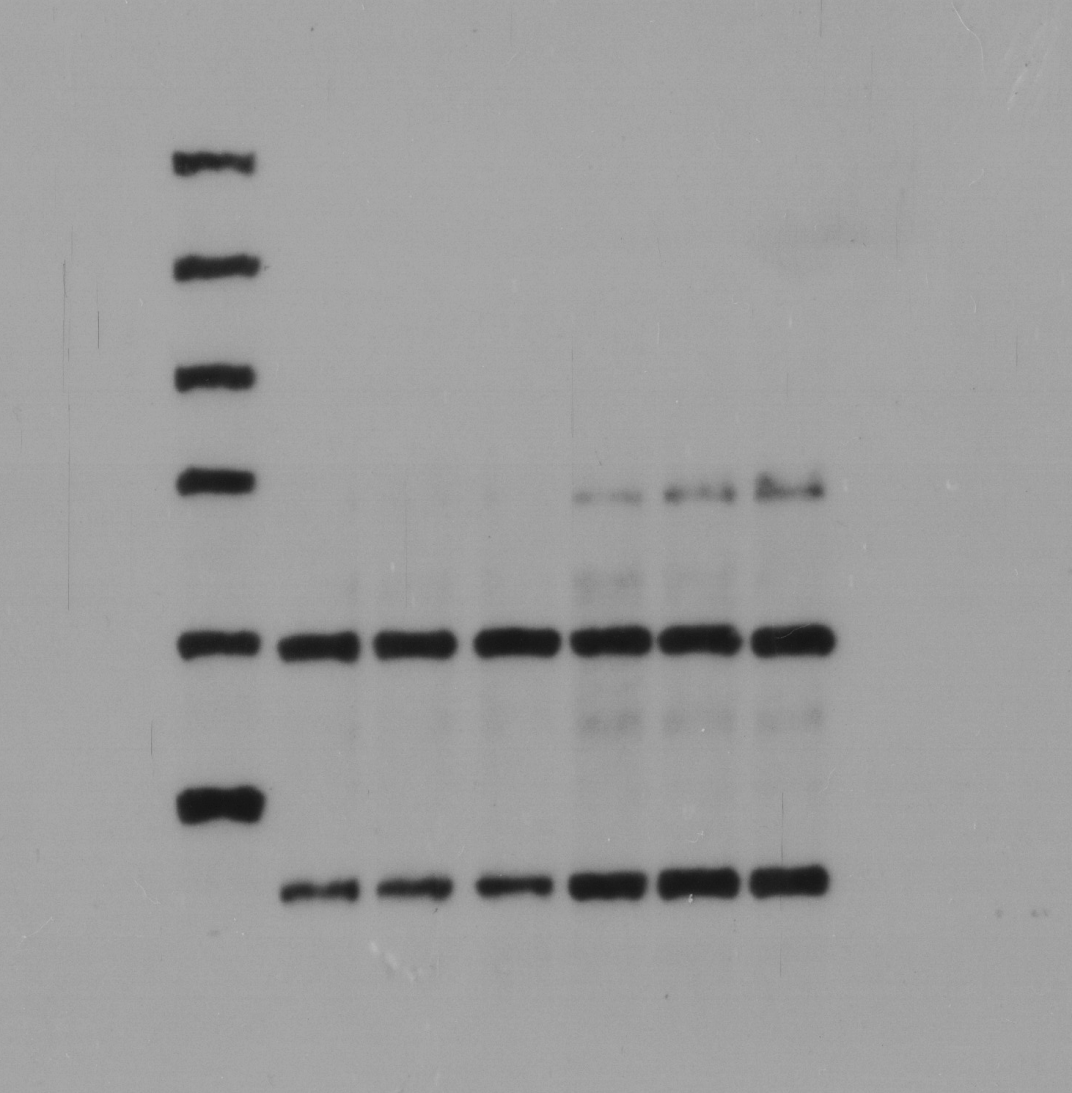


GABARAP

(14KDA)

GAPDH

(37KDA)

Sham

Model

2、ITCH (A17389, Abclonal, 1/1000）；β-tubulin (T0023, Affinity, 1/8000)


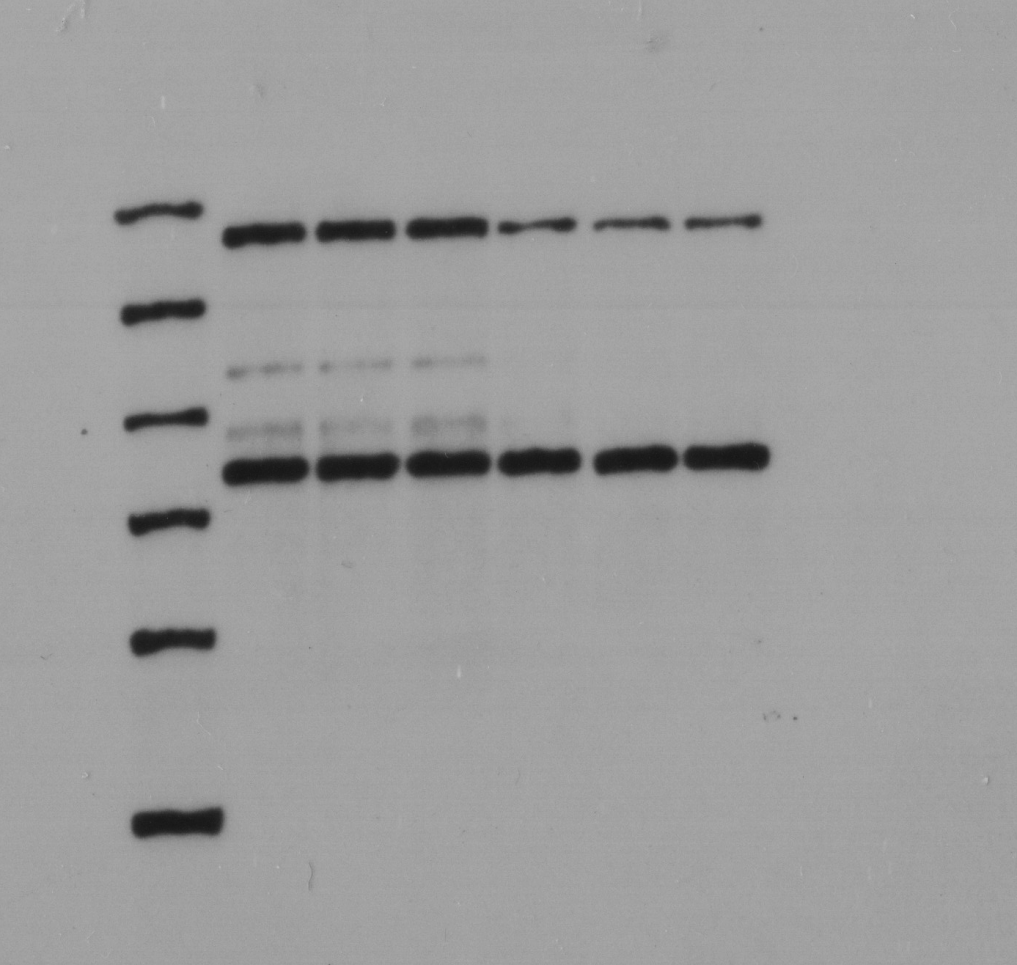


β-tubulin

(55KDA)

ITCH

(100KDA)

Model

Sham

3、Marker molecular weight diagram (AP13L092, Life-ilab)


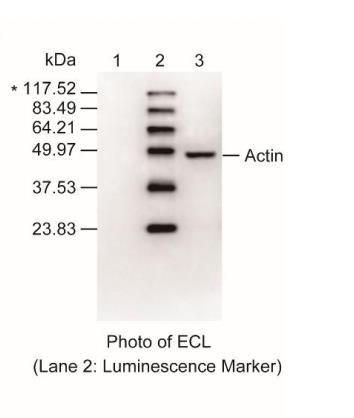

Supplement: Supplementary file 3 — Supplementary Material 3 [file 41065_2025_522_MOESM3_ESM.docx]
